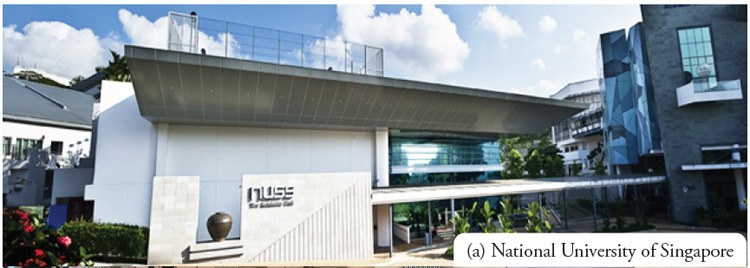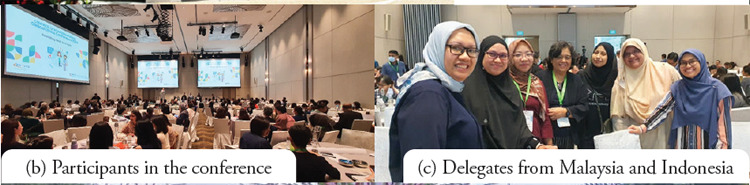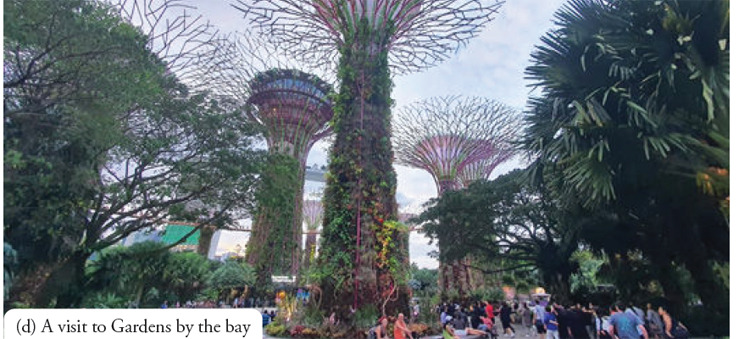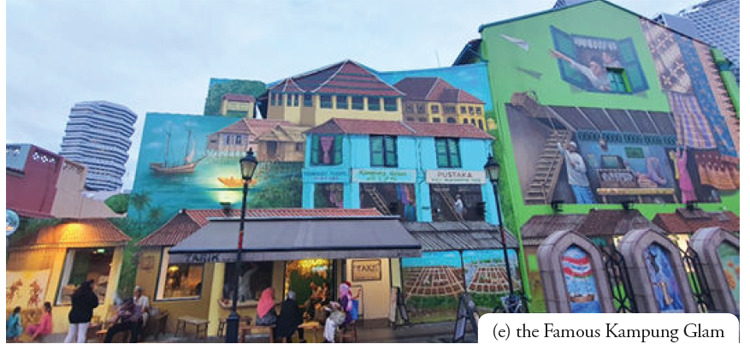# My journey in Medical Ethics: Reflecting on the Singapore Medical Ethics Conference

**DOI:** 10.51866/mol.6l8

**Published:** 2024-03-19

**Authors:** Abu Hassan Hasliza

**Affiliations:** 1 MBBS, MMed (Family Medicine), Department of Primary Care Medicine, Faculty of Medicine and Defence Health, National Defence University of Malaysia, Kuala Lumpur, Malaysia. Email: hasliza@upnm.edu.my

**Keywords:** Medical ethics, Conference, knowledge, Best interests

In January 2024, I had the privilege of experiencing the inspirational atmosphere of the Centre for Biomedical Ethics at the National University of Singapore (NUS). During my time at NUS, I had the opportunity to immerse myself in the captivating realm of the CENTRES Clinical Ethics Conference 2024, focused on the theme "Revisiting Best Interests." The two day event offered abundant opportunity for networking, discussion and knowledge exchange through lectures, quizzes, and engaging sessions with experts in medical ethics and law, both locally and internationally.

The aim of the conference is to equip healthcare professionals with a deeper understanding of the multifaceted concept of best interests across various contexts, including patient welfare, beneficence, capacity, and mental illness. The expansive scope of best interests enables me to address complex issues and cases during discussions, offering flexibility in the approach. As a primary care physician managing patients from diverse backgrounds, I frequently encounter tough decisions regarding their best interests. This conference sheds light on my journey in medical ethics and jurisprudence, enhancing my grasp of medical ethics concepts and clarifying the principle of best interest.

The sessions were categorized into four groups, involving:

**Session 1: Vulnerable Adults**

Lectures delivered by physicians and experts in medical ethics focussing on the evaluation of best interests in this population. The discussion involved a varied set of patients, including those with dementia, memory problems, mental health issues, and those facing abuse.

**Session 2: Persons with Psychiatric Issues**

A productive evening was spent discussing how much importance should be placed on the preferences and values of individuals with mental illness when deciding if a treatment is in their best interests. The session delved into the ethical aspects of covert medication and examined whether it presents an ethical choice or a moral hazard using a case-based scenario.

**Session 3: Persons at the End-of-Life**

On the conference's second day, the focus was on exploring unwise patient preferences and determining what is in their best interest. Decision-making for incapacitated individuals at the end of their life involves a distinct set of guiding factors and complexities. This includes considering whether their wishes and desires regarding care were expressed beforehand. We discussed about making choices that are best for the patients and looking at what the healthcare team, family, and legal representatives need to do.

**Session 4: Minors**

One of the topics I particularly enjoyed was about determining the best interests of minors. Identifying what is best for a child can be challenging, especially when there are conflicts between the medical team and the parents. The session emphasized that in these cases, there could be doubts about how much parents should be involved, which might result in seeking advice from the Courts to decide what's best for the child.

In conclusion, I have learned a lot more about medical ethics and I was especially enlightened by the principle of current best interests. This knowledge acquired is undoubtedly highly applicable in my daily clinical practice.

The images below show some information about the conference and some of the activities which I have participated in while I was in the conference in Singapore.

**Figure:**